# Strengthening the Application of the DAIDS GCLP Guidelines: The Implementation of an Integrated Laboratory Oversight Framework

**DOI:** 10.1089/aid.2024.0042

**Published:** 2024-11-06

**Authors:** Loc Nguyen, Anne Leach, Estelle Piwowar-Manning, Mark Marzinke, Allan Levesque, Claudine Gregorio, Kristen Skinner, Tiri Towindo, Heidi Hanes, Kwabena Sarpong, Christian Kasongo, Natasha Samsunder, Grace Aldrovandi, Kathie G. Ferbas, Andries Engelbrecht, Michael Stirewalt, Emily Anyango, Sasiwimol Ubolyam, Pamela Lankford-Turner, Marcella Sarzotti-Kelsoe

**Affiliations:** ^1^ PPD, Part of Thermo Fisher Scientific, Wilmington, North Carolina, USA.; ^2^ Patient Safety Monitoring in International Laboratories (pSMILE), Department of Pathology, Johns Hopkins University, Baltimore, Maryland, USA.; ^3^ HIV Prevention Trials Network LC, Department of Pathology, Johns Hopkins University, Baltimore, Maryland, USA.; ^4^ Westat, Rockville, Maryland, USA.; ^5^ Department of Surgery and Center for AIDS Research, Duke University Medical Center, Durham, North Carolina, USA.; ^6^ Infectious Diseases Institute (IDI) Core Lab, Kampala, Uganda.; ^7^ The Aurum Institute, Johannesburg, South Africa.; ^8^ Centre for the AIDS Programme of Research in South Africa (CAPRISA), Berea, South Africa.; ^9^ International Maternal Pediatric Adolescent AIDS Clinical Trials and AIDS Clinical Trials Group, University of California, Los Angeles, California, USA.; ^10^ HIV Vaccine Trials Network LC, Cape Town, South Africa.; ^11^ Kenya Medical Research Institute, Nairobi, Kenya.; ^12^ HIV Netherlands Australia Thailand (HIV-NAT) AIDS Research Centre, Bangkok, Thailand.; ^13^ Emory Infectious Diseases Clinic Research Laboratory, Decatur, Georgia.; ^14^ HIV Vaccine Trials Network LC, Seattle, Washington, USA.

**Keywords:** Clinical Trials, Collaboration, DAIDS GCLP Guidelines, GCLP training, GCLP audit, Laboratory Quality Improvement

## Abstract

The Division of AIDS (DAIDS) Good Clinical Laboratory Practice (GCLP) Guidelines establish a framework to guide the oversight of laboratories supporting DAIDS-sponsored clinical research or trials. Compliance with these guidelines promotes data reliability, validity, and safety of the clinical research or trial participants and laboratory staff and ensures adherence to regulatory requirements. Acknowledgment and adoption of the DAIDS GCLP Guidelines are critical in building laboratory capacity and preparedness for conducting clinical trials. In collaboration with DAIDS, laboratory experts support the implementation of the DAIDS Integrated Laboratory Oversight Framework (Framework) activities. This article describes the implementation of the GCLP Guidelines, the Framework activities, and the coordinated efforts to strengthen laboratory performance. The Framework activities include four components: Quality Assurance Oversight, GCLP Audits, GCLP Training, and Laboratory Quality Improvement. Comparison of GCLP Guidelines with other regulations or standards, including U.S. Clinical Laboratory Improvement Amendments regulation 42 CFR 493, College of American Pathologists, World Health Organization GCLP, and International Organization for Standardization, ISO 15189:2012 standards, highlighted the differences and similarities to guide integration and harmonization efforts. Processes related to the Framework activities are outlined in detail, including key data derived from the managed activities of over 175 laboratories worldwide. Via the evolution of the DAIDS GCLP Guidelines and laboratory oversight workflows, the laboratories participating in DAIDS-sponsored clinical research and trials have successfully participated in internal and external regulatory audits. The collaborative and integrated oversight approach promotes knowledge-sharing and accountability to support the implementation of the DAIDS GCLP Guidelines and compliance monitoring. Lessons learned have helped with the implementation of the DAIDS integrated laboratory oversight approach and quality oversight programs at multiple laboratories worldwide.

## Introduction

Within the Division of AIDS (DAIDS), laboratory oversight is guided by the DAIDS Good Clinical Laboratory Practice (GCLP) Guidelines^1^ following an Integrated Laboratory Oversight Framework (Framework), which integrates expertise from various partners. The Framework activities include four components: Quality Assurance (QA) Oversight, GCLP Training, GCLP Audits, and Laboratory Quality Improvement. The DAIDS Clinical Laboratory Oversight Team (DCLOT),^2^ representing DAIDS (Sponsor), with support from the Collaborative Laboratory Oversight Team (Oversight Team) oversees the application and implementation of the DAIDS GCLP Guidelines (hereafter referred to as the “GCLP Guidelines”) and supporting activities. The Oversight Team comprises laboratory experts representing DCLOT, DAIDS Clinical Research Networks (Networks), DAIDS auditing contractors, and QA contractors.^3^ Collaborative activities under the purview of the Oversight Team include developing policies and procedures to oversee over 175 laboratories in the United States and non-U.S. settings.^4^

A collaborative partnership is essential to establish an integrated team of laboratory experts and apply a robust risk-based oversight framework. The application of the GCLP Guidelines from the Sponsor’s perspective addressing the collaborative implementation and evaluation efforts, as well as challenges and opportunities for improvement, is described in a complementary article.^3^ The overarching goal of the Oversight Team is to set the requirements and recommendations to ensure the robustness of laboratory testing in various clinical laboratory environments. In the United States, Clinical Laboratory Improvement Amendments (CLIA)^5^ establish quality standards for clinical laboratories; hence, CLIA-compliant laboratories are exempt from GCLP compliance. This article describes the implementation of the collaborative approach using the Framework and input from the Oversight Team. The implementation of the GCLP Guidelines, the partners’ perspective, and the evaluation of efficacy, including challenges and opportunities for improvement, are described.

## Implementation of the DAIDS GCLP Guidelines

The implementation of the GCLP Guidelines requires the Oversight Team to apply processes and procedures within their functional activities to ensure laboratory compliance with such guidelines. Adherence to GCLP Guidelines enables laboratories to generate accurate and reliable data, which facilitates data sharing and comparison across different laboratories and studies. The GCLP Guidelines reflect clinical laboratory operation requirements and support the comparability of data generated in multisite clinical research or trials. The GCLP Guidelines incorporate sections of standards and guidelines from other accrediting bodies, offering geographically and scientifically broader applicability.^1,4^

The GCLP Guidelines were compared with those from Good Laboratory Practice (GLP; 21 CFR 58), CLIA, College of American Pathologists (CAP), International Organization for Standardization (ISO 15189:2012), European Medicines Agency (EMA), Good Clinical Laboratory Practices World Health Organization (WHO/GCLP), and South African National Accreditation System (SANAS).^1,4–7^ The GCLP Guidelines, version 4.1 published in 2021, align better with current regulatory requirements and recommendations promoted by other groups (GLP, CLIA, CAP, ISO 15189:2012, EMA, WHO/GCLP, and SANAS) to ensure the generation of high-quality results in support of clinical research or trials. Similarities and differences in Key Laboratory Operational Topics are described below and summarized in Table 1.

**Table 1.** Comparing DAIDS GCLP Guidelines—Key Similarities and Differences

	GLP	CLIA	CAP	ISO 15189:2012	EMA GCLP	WHO	SANAS
Organization and personnel	Equivalent	Stricter requirements	Checklist defining roster roles requirement	Requires effectiveness check	Equivalent	Equivalent	Requires effectiveness check
Equipment	Equivalent	Equivalent	Equivalent	Purchasing Decontamination procedures required	Equivalent	Equivalent	Purchasing Decontamination procedures required
Testing facility operation	Literature may be used to supplement SOP	Literature may be used to supplement SOP	Delayed testing needs notification	Hand editing is allowed until reissue; all documentation subject to Doc Control	Equivalent	Supplemental material for SOPs must be retained; no frequency of review dictated	Hand editing is allowed until reissue all documentation subject to Doc Control
Test and control	Equivalent	Equivalent with additional labeling requirements	Equivalent	Equivalent	Equivalent	Equivalent	Equivalent
Test method validation and verification	No reference available	Equivalent	Validation/verification requirements for FDA modified and LDT	Requires measurement of uncertainty	Equivalent	Equivalent	Requires measurement of uncertainty
Records and reports	Mandates specific retention requirements	Mandates specific retention requirements	Mandates specific retention requirements	Equivalent	Equivalent	Equivalent	Equivalent
Physical facilities	Separate laboratory spaces	Equivalent	Separate laboratory spaces	Equivalent	Equivalent	Equivalent	Equivalent
Specimen transport and management	No reference available	Equivalent	Equivalent	Equivalent	Equivalent	Equivalent	Equivalent
Personnel safety	Protection of test systems	Comply with local standards	Comply with local standards	Equivalent	No reference available	Comply with local standards	Equivalent
Laboratory information systems	Equivalent	Equivalent	Equivalent	Equivalent	Equivalent	Equivalent	Equivalent
Quality management	Equivalent	Equivalent	Equivalent	KQI priority based on risk	Documented procedures required referral laboratories	Audits, audit certificate	Documented procedures required referral laboratories

CAP, College of American Pathologists; CLIA, Clinical Laboratory Improvement Amendments; DAIDS, Division of AIDS; EMA, European Medicines Agency; FDA, U.S. Food and Drug Administration; GCLP, Good Clinical Laboratory Practice; GLP, Good Laboratory Practice; ISO, International Organization for Standardization; LDT, Laboratory Developed Test; LIS, laboratory information systems; SANAS, South African National Accreditation System; SOP, standard operating procedure; WHO, World Health Organization.

- Organization and personnel: Aligns well with other regulatory standards, although other standards (CAP, CLIA, ISO 15189:2012, and SANAS) have additional requirements for personnel and training program effectiveness.
- Equipment: Aligns well with two standards regarding the need for equipment purchasing, standard operating procedures (SOPs), and documentation of equipment decontamination.
- Testing facility operation: Aligns well with other standards. There are allowances for using supplemental material and notification of testing delays.
- Test and control: Aligns well with all other agencies.
- Test method validation and verification: Aligns well with other standards; however, ISO 15189:2012 and SANAS further require the establishment of measurement of uncertainty.
- Records and reports: While there were no significant differences between standards, the duration for which clinical trial documents should be retained can vary depending on the requirements set forth by Sponsors, regulatory authorities, local authorities, and institutional policies.
- Physical facilities: Aligns well with other standards, although some standards indicate the need to have distinct laboratory spaces and specific activities.
- Specimen transport and management: Aims to ensure the proper transport and management of specimens in DAIDS-sponsored clinical research or trials, emphasizing the importance of specimen integrity, traceability, and compliance with regulatory requirements. These requirements are similar across other standards.
- Personnel safety: Aligns with other standards, focusing on the well-being and safety of laboratory personnel.
- Laboratory information systems (LIS): All mentioned guidelines and standards have similar approaches to GCLP Guidelines, outlining requirements for LIS to ensure proper management and documentation of laboratory data including data management, data integrity and security, electronic data capture, data review and approval, data retrieval and reporting, and training and documentation.
- Quality management: Aligns well with other agency standards. EMA requires that management has a documented procedure for selecting and evaluating referral laboratories. ISO 15189:2012 emphasizes continual improvement activities directed at areas of the highest priority based on risk assessments. Reporting to Management, new in GCLP version 4.1, is consistent with that found in other standards.

GCLP compliance ensures all documents related to clinical studies are well maintained and the participating laboratories generate reliable results, which assures trust from the Sponsor. GCLP compliance further assists with standardizing laboratory processes across the different clinical research or trial sites for a protocol.

## The DAIDS Framework

The Framework outlines a collaborative approach by the Oversight Team to implement and monitor laboratory quality activities.

### QA Oversight

**FIG. 1.**
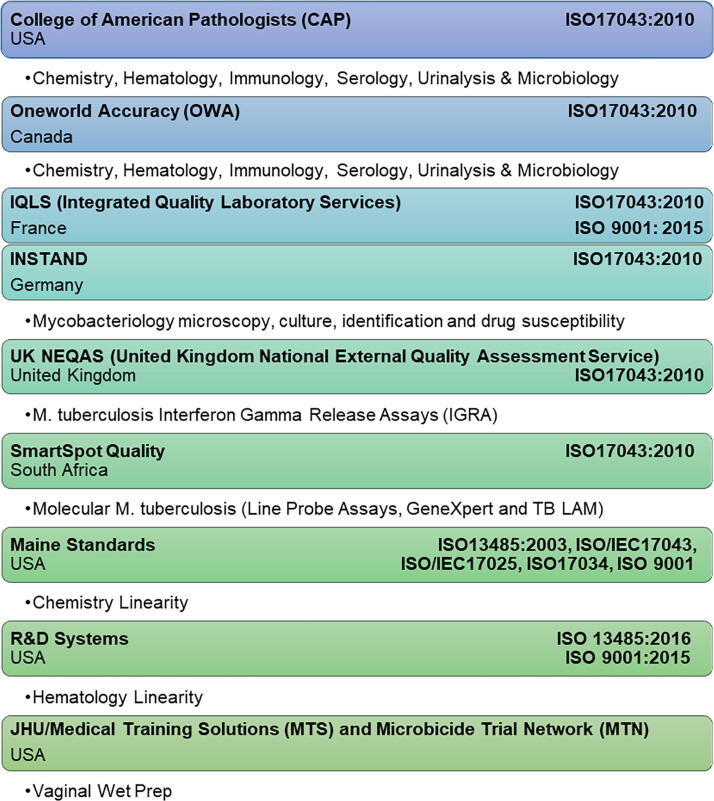
The figure includes a listing of commercial external quality assurance (EQA) providers used by the Division of AIDS Quality Assurance contractor to provide a customized approach to ensure the reliability of protocol-related tests. The bulleted text below each provider details the types of assays/methods/analytes tested for each. Also included in the table is the International Organization for Standardization accreditation obtained by each EQA provider.

QA Oversight is a method for evaluating and ensuring adherence to quality standards throughout clinical research or trial.^4^ A key element of QA Oversight is the implementation of external quality assurance (EQA) or proficiency testing (PT); EQA or PT is used interchangeably here. EQA is a quantitative measure of laboratory quality and provides an external, unbiased confirmation that a laboratory can produce reliable results. It is a requirement by most regulators or accrediting bodies for any testing other than CLIA-waived testing. Waived tests include test systems cleared by the U.S. Food and Drug Administration (FDA) for home use and those tests approved for waiver under the CLIA criteria. Typically, EQA involves testing blinded samples obtained from a third party. EQA panel samples are treated as participant samples and run by personnel who routinely perform the testing. This ensures the reliability of testing by laboratory personnel. Results are submitted to the EQA provider for evaluation which is typically performed by statistical comparison to peer data or known values. Commercial EQA programs are valuable only when correctly aligned to the method, the analyte, the peer group, and the needs of the study protocol. DAIDS QA contractors utilize a customized approach to ensure accurate and reliable EQA coverage.^8^ A list of the various commercial EQA providers currently used may be found in Figure 1.

Commercial EQA programs may sometimes be unavailable for protocol-specific assays. In those cases, the laboratory must have a plan for an alternative assay performance assessment approved by laboratory management, using interlaboratory comparison or self-blinded samples. The number of laboratories performing protocol-specific assays in HIV/AIDS clinical trial specimens expanded in size over the past 20 years of testing. Therefore, multiple noncommercial EQA programs were created to fill the need for standardized PT comparisons. A nonexhaustive series of such EQA programs is presented here. Among them is the Virology Quality Assurance (VQA) program, funded through the National Institute of Allergy and Infectious Diseases (NIAID).^9^ The VQA program provides comprehensive EQA assessment for laboratories doing virological testing targeting HIV used in NIAID-supported clinical trials. The NIAID VQA program has been in operation since 1988. Another example of such interlaboratory comparison is the immunology quality assessment (IQA) program, which statistically assesses proficiency in T-cell subset measurement (CD3(+)4(+)/CD3(+)8(+) percentages and absolute counts) for each laboratory supporting the NIAID DAIDS HIV clinical trial networks.^10^ A longitudinal analysis of T-cell subset measurement PT was performed between January 2003 and July 2012.^10^ The analyzed results demonstrated that proficiency within and between IQA laboratories improved over time, with differences noticed when single versus dual platform flow cytometry technologies were employed.

Another example of specialized interlaboratory statistical comparison is the External Quality Assurance Program Oversight Laboratory (EQAPOL), also funded by the NIAID-DAIDS.^11–14^ EQAPOL supports the oversight of EQA programs that monitor laboratories involved in HIV/AIDS research and vaccine trials worldwide. EQAPOL includes four EQA programs assessing proficiency in the following assays: interferon-gamma, enzyme-linked immunosorbent spot (ELISpot) assay, intracellular cytokine staining by flow cytometry, Luminex/multiplex bead-based assay, and HIV incidence assay testing.^11–14^ GCLP compliance is required and consistently assessed at the four EQAPOL oversight laboratories administering the EQA programs, at the IQA central oversight laboratory, and at the central biorepository laboratory, Immunology Virology Quality Assessment Center, preparing, storing, and shipping the PT kits to the testing laboratories.^15^

A further effective example of an EQA program for specialized endpoint assays was developed to ensure data equivalency across laboratories performing HIV-1 neutralizing antibody assays in TZM-bl cells, for HIV/AIDS vaccine clinical trials (T-PTP).^16^ This EQA program is the first available for the evaluation and documentation of assay equivalency for laboratories performing such endpoint assay and may provide guidance for the development of future proficiency testing programs for other assay platforms. The T-PTP is supported by NIAID-DAIDS and the Gates-funded Collaboration for AIDS Vaccine Discovery.^16^ Interestingly, in preliminary results from the analysis of the T-PTP program, it was noticed that a greater number of failed results occurred in participating laboratories that were not audited for GCLP compliance.

Overall, the laboratory’s participation in PT programs provides many opportunities to ensure accuracy, support laboratory improvement, and troubleshoot equipment-, operator- and test reagent-related problems. The GCLP Guidelines provide direction to laboratories on using EQA as part of the overarching quality management program. Laboratories participating in DAIDS-sponsored clinical research or trials must enroll in DAIDS-approved EQA programs that cover all protocol analytes. When commercially prepared EQA is not available, the QA contractors, in collaboration with the sponsor, work to ensure that all methods are covered by utilizing novel technologies, virtual panels, or customized PT programs developed and monitored by the QA contractors.

Laboratory performance in DAIDS-approved PT programs is monitored and reviewed monthly by DAIDS and the Networks. Any unsuccessful EQA must be investigated to determine the root cause of the failure. The QA contractors work with the laboratories on the investigation and with the Networks providing their input and approval of the outcome of the investigation and corrective action. The Sponsor documents a final acknowledgment that the investigation is complete. It is important to note that the EQA provider assesses the success of each EQA challenge. It is up to the Sponsor and the Networks to determine how the EQA data are used.

By providing this customized level of assistance, the laboratory is guided and trained to improve its processes. EQA testing also provides warning signals for systematic problems and objective evidence of testing quality and identifies areas for improvement or enhanced training. EQA, as part of the GCLP framework, has been proven to improve laboratory performance as evidenced by several publications.^17–19^ The use of standardized, commercial, and noncommercial EQA panels resulted in sustained improvement in analyte performance over time.^17^ Applying approved EQA also assists with comparing results generated across sites for protocol data analysis.

### GCLP Training

To facilitate the incorporation of GCLP principles into laboratory operations, DAIDS also supports GCLP compliance through the provision of various GCLP Training programs, including online training through the GCLP e-learning modules and face-to-face GCLP Training.^4^

**FIG. 2.**
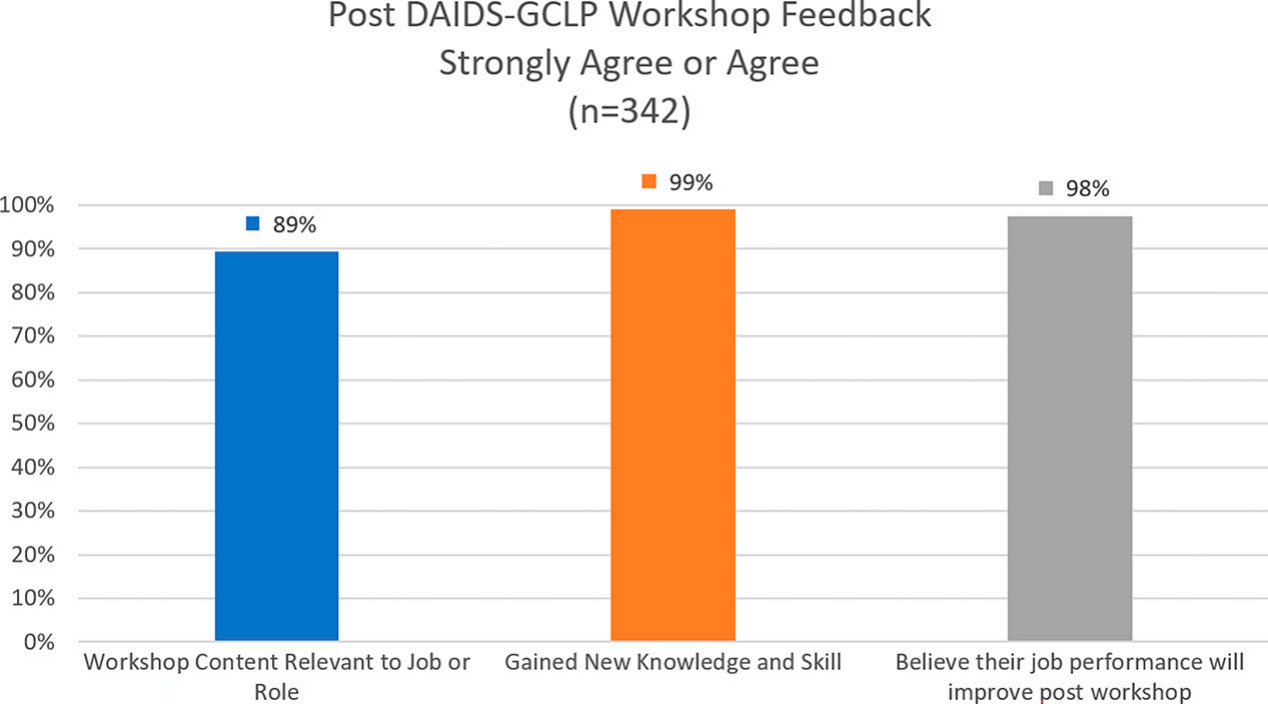
The schematic diagram illustrates a clustered column chart with average agreement percentages of post-face-to-face Good Clinical Laboratory Practice training feedback aggregated from 342 respondents in 6 events conducted in 2023. Percentage of agreement for workshop content relevant to job or role is shown in *blue*. Percentage of agreement for new knowledge or skills gained from the workshop is shown in *orange*. The percentage of agreement that the workshop will contribute to their job performance is shown in *gray*.

GCLP Training programs impart knowledge, skills, and tools required for the application of the Guidelines into the laboratory’s quality management system. The GCLP e-learning modules provide an interactive platform with voice-over narratives, activities, scenarios, photos, quizzes, multiple choice, true-false, and match-up questions and answers. The three-day comprehensive face-to-face GCLP Training workshops held regionally in Asia, Africa, and Latin America bring laboratory professionals from different countries into a single setting, facilitating the sharing of ideas, challenges, and best practices. Post-face-to-face GCLP Training feedback aggregated from 342 respondents in 6 separate events conducted in 2023 showed significant agreement that the contents presented were relevant (89%) and that the participants gained new knowledge or skills (99%) and believed that their job performance will improve after workshop (98%) (Fig. 2).

Positive feedback from such events has revealed the crucial role that GCLP Training plays in ensuring the need for clinical research laboratories to generate quality data that can be used effectively for clinical trial safety and efficacy analyses.

#### Key benefits of DAIDS-sponsored GCLP Training

##### Enhanced compliance

GCLP Training helps laboratories understand and comply with the specific requirements and guidelines set forth by DAIDS. It ensures that laboratories meet the necessary standards, protocols, and quality control measures, promoting compliance with regulatory and funding agency expectations.

##### Improved quality

GCLP Training emphasizes quality management principles and best practices in laboratory operations. It equips laboratory staff with the knowledge and skills to implement quality control measures, follow standard operating procedures, and maintain accurate and reliable data. This leads to improved overall quality in laboratory practices and data generated.

##### Standardization of processes

GCLP Training promotes standardized laboratory processes across laboratories. It ensures that laboratories involved in DAIDS-sponsored clinical research or trials follow consistent protocols, methodologies, and quality control measures. This standardization allows for the comparability of data and results across multiple research sites, enhancing the reliability and validity of findings.

##### Increased data integrity

GCLP training emphasizes the importance of data integrity in laboratory processes. It provides guidelines and best practices for data collection, handling, storage, and analysis, ensuring the accuracy, completeness, and reliability of research data.^1^ This enhances the credibility of study outcomes and supports evidence-based decision-making.

##### Participant safety

GCLP training includes a focus on participant safety in laboratory operations. It educates laboratory staff on appropriate biosafety practices, proper handling of biological specimens, and adherence to safety protocols. This helps minimize risks to participants and ensures the ethical conduct of research.

##### Professional development

GCLP training offers professional development opportunities for laboratory personnel. It enhances their knowledge, skills, and competence in laboratory practices, quality management, and regulatory compliance. This benefits the laboratory and contributes to professional growth and career advancements.

##### Collaboration and networking

GCLP training programs often facilitate collaboration and networking among laboratories involved in DAIDS-sponsored clinical research or trials. They provide opportunities for knowledge sharing, exchange of experiences, and building professional relationships with peers and experts in the field. This promotes a supportive research community and fosters collaboration in advancing HIV/AIDS research. This also allows the laboratory teams to gain confidence in the quality and be comfortable enough with the regulations to speak with authority on any discrepancies or disagreements, which helps to strengthen the overall laboratory quality. Criteria for selecting GCLP trainers are based on proficiency with GCLP concepts gained from working in a GCLP-compliant laboratory, conducting GCLP Audits, and GCLP Training experience. From a network perspective, the Network staff undergoes GCLP Training at a minimum when the Guidelines are updated or on an as-needed basis. This allows Network staff to understand and incorporate the Guidelines to assist laboratories.

In summary, DAIDS-sponsored GCLP Training provides laboratories with enhanced compliance, improved quality, standardized practices, increased data integrity, participant safety, professional development opportunities, funding eligibility, and collaboration benefits. These benefits contribute to the overall success and impact of HIV/AIDS research conducted by laboratories involved in DAIDS-sponsored projects.

### GCLP Audits

The applicable GCLP Audits are typically conducted once per year for each laboratory, utilizing approved audit checklist templates appropriate to the targeted laboratory. The audit checklist templates are listed in order of use frequency: general laboratory, Peripheral Blood Mononuclear Cells, tuberculosis, central laboratory, and biorepository.^20^

*Ad hoc* audits (e.g., for-cause inspections) are also performed when needed. Besides laboratory audits, QA auditors and DCLOT staff may be involved in the *ad hoc* audits, and these parties, along with Network staff, can provide assistance to the laboratory.^4^ Participation in DAIDS GCLP audits can ensure compliance, improve quality, enhance data integrity, mitigate risks, drive process improvement, and support training and education. DAIDS GCLP Audits provide several benefits to laboratories involved in DAIDS-sponsored clinical research or trials:

- Compliance verification: The audits verify whether the laboratory is compliant with DAIDS GCLP guidelines. Audits assess the laboratory’s adherence to the required standards, protocols, and quality control measures. This helps the laboratory ensure that it is meeting the necessary regulatory and quality requirements for conducting research.
- Quality assurance: Audits support quality assurance efforts within the laboratory. Audits evaluate the laboratory’s processes, procedures, and documentation to meet the required standards. By identifying areas of improvement or noncompliance, audits enable the laboratory to implement corrective actions and enhance its overall quality management system.
- Data integrity and reliability: Audits focus on data integrity and reliability. Audits review the laboratory’s data management practices, including data collection, handling, storage, and analysis. This ensures that data generated by the laboratory are accurate, complete, and reliable, which is essential for producing high-quality research outcomes.
- Risk identification and mitigation: GCLP Audits help laboratories identify and mitigate risks associated with their operations. Audits assess potential risks impacting participant safety, sample integrity, or data quality. By identifying these risks, audits enable the laboratory to implement risk mitigation strategies, such as improving processes, enhancing training, or implementing additional quality control measures.
- Process improvement: GCLP Audits provide an opportunity for process improvement within the laboratory. Audits identify areas where the laboratory can enhance its practices, procedures, and documentation to align with the GCLP Guidelines. This promotes continuous improvement and helps the laboratory optimize its operations for greater efficiency, accuracy, and compliance.
- Training and education: GCLP Audits help identify training and educational needs within the laboratory. Audits may highlight areas where additional training or education is required to improve personnel competence and ensure adherence to the GCLP Guidelines. This supports professional development and helps maintain a skilled and knowledgeable workforce within the laboratory.

Once the GCLP Audit is completed, the auditing contractor provides an audit report to the DCLOT representative and DAIDS QA contractor. DAIDS QA contractor then works to create an action plan (AP) based on the findings in the audit report. AP items are derived by comparing the comments in the audit report with the GCLP standards and determining if the laboratory is out of compliance with GCLP Guidelines. DAIDS QA contractor also provides suggested actions for remediation of the finding.

The Networks and eDCLOT review the AP before distribution to the laboratory. Network representatives review the audit report along with the action items generated to ensure accuracy. The AP is reviewed to determine grading and designate the findings as Critical, Major, Minor, or Recommended as per the GCLP. Network representative consider the number of findings, severity, and recurrent findings and use the information as one of the key quality indicators (KQI) when determining laboratory risk assessments.

The AP is then distributed to the laboratory and a timeline for completion is requested. Once the laboratory submits remediation documentation, the QA contractor works with the laboratory to close action items and store documentation. The Networks also monitor AP resolution providing support and assistance to the laboratories in resolving findings when warranted. The networks will review closed APs to ensure that the item closed is appropriate for the network and works with the site as needed on network-specific action items. An Audit Participation Certificate is issued by the auditor, a recent improvement of the DAIDS GCLP Audit program, advocated in the past by several GCLP subject matter experts.

### Laboratory Quality Improvement

The Laboratory Quality Improvement component encompasses monitoring laboratory performance and collaborative partner activities by the Sponsor.^4^ Quality improvement starts with a commitment from the laboratory management to the laboratory bench. Recognizing situations where quality may be lacking, a close look is needed to determine the “why.” This can be a result of systems, procedures, training, and, on occasion, human error—that is, perhaps too much pressure to get the task completed, a lack of understanding of what needs to be done, too many procedural changes over time, and, unfortunately, there are times when the individual chosen may not be the most suitable candidate for the task at hand. When working for multiple entities, there can be subtle differences in how each entity wants the laboratory work done; this can also complicate quality. The pandemic educated us that supply chains, delivery, and even internal staffing can be challenging; so planning to meet laboratory needs will lead to quality improvement.

Tracking quality issues can also be useful for managing and enhancing quality improvement. The development, trending, and review of site-specific KQI is a critical component, recently refined in GCLP, version 4.1, which emphasizes the establishment of KQI “benchmarks,” their annual appraisal of effectiveness, and their integration into the Quality Management  program. KQIs and their associated benchmarks can be developed to focus on topics such as laboratory personnel development and training, re-education of staff to improve knowledge about GCLP Guidelines, EQA performance, and adherence to site-specific SOPs, thus reducing the rate of protocol deviations. The DAIDS e-learning portal serves as a key resource for laboratories supporting DAIDS clinical research or trials. The DAIDS GCLP sets a standard for quality, and participation in the program enables laboratories to continually move up the quality scale.

## Conclusion

### Lessons learned

The success and quality improvement of DAIDS-sponsored laboratories underscore the robustness of the GCLP Guidelines. These Guidelines are characterized by clarity, offering laboratories specific guidance rather than mere options or suggestions. GCLP stands out among other laboratory standards because it provides a more structured framework, including mandatory elements and minimum requirements. The strength of GCLP is further emphasized through collaboration and feedback from partners, particularly evident during the revision to version 4.1, which involved the inclusive participation of all partners in the drafting update. Further collaboration is evident through the success of the DAIDS e-learning portal, serving as a major source for laboratories supporting DAIDS clinical research or trials. The successful implementation of laboratory oversight is achieved through a collaborative approach, where all partners work together with the sponsor to achieve laboratory quality.

### Challenges and opportunities

The reciprocal relationship between GCLP compliance and accreditation to the CAP and ISO 15189:2012 standards is crucial to note. Despite slight variations in standards, the shared mindset needed to comply with standards and undergo audits facilitates a seamless process. Laboratories actively engage in collaborative discussions to determine and monitor quality indicators and benchmarks, providing tangible evidence of ongoing improvement. However, it is essential to emphasize that there is no GCLP “light”—it is a total quality improvement package. A laboratory cannot claim GCLP compliance until an independent auditor has inspected it according to the GCLP guidelines.

### Strengthening Laboratory Performance Improvement

The implementation of GCLP not only offers a fertile platform for laboratories aspiring for CAP or ISO 15189:2012 accreditation but also raises the quality of all laboratory operations, going beyond those directly linked to DAIDS-sponsored research and trials. The DAIDS GCLP sets a standard for quality, and the program continues to move laboratories up the quality scale.
